# Peptone Enemata

**Published:** 1887-07

**Authors:** 


PEPTONE ENEMATA.—Ewald urges the more general introduction of peptone enemata. His researches have led him to conclude:

1. The power of the rectum to absorb is undoubted; the amounts, however, which are absorbed vary greatly from influences not to be controlled and peculiar to the individual, so that a purely physica or chemical action, independent of nervous influence, which can be produced by the necessary means at will, does not exist.

2. The appropriateness of an albuminoid for rectal absorption is not dependent upon its richness in genuine peptone. Eggs which contain the smallest quantity of peptone are as readily absorbed, and even give a greater gain to the organism than peptones which have a double or quintuple amount of contained peptone.

3. We are able to produce with unprepared eggs, and still better after preparation with hydrochloric acid and pepsin, the same results which are obtained with purchased peptones, and at about one-halt the cost.—*Therap. Monatshefte*, No. 3, 1887.

